# Current Approaches to the Management of Postoperative Fistulas in Gastric Cancer Surgery: Experience of a Tertiary Center

**DOI:** 10.3390/jcm14051733

**Published:** 2025-03-04

**Authors:** Alin Fetti, Roxana Zaharie, Vlad Radu Puia, Dan Valean, Roman Taulean, Vlad Nechita, Florin Zaharie, Ioan Catalin Bodea, Oana Moșincat, Nadim Al-Hajjar

**Affiliations:** 1 Regional Institute of Gastroenterology and Hepatology “Octavian Fodor”, 400162 Cluj-Napoca, Romania; dr.alinfetti@yahoo.com (A.F.); valean.d92@gmail.com (D.V.); axiromplus@gmail.com (R.T.); nechita.vlad@umfcluj.ro (V.N.); bodea_cata@yahoo.com (I.C.B.); mosincat.oana.maria@elearn.umfcluj.ro (O.M.); na_hajjar@yahoo.com (N.A.-H.); 2 Department of General Surgery, University of Medicine and Pharmacy “Iuliu Hațieganu”, 400347 Cluj-Napoca, Romania; 3 Department of Gastroenterology, University of Medicine and Pharmacy “Iuliu Hațieganu”, 400347 Cluj-Napoca, Romania

**Keywords:** gastric cancer, anastomotic leak, postoperative management, minimally invasive surgery

## Abstract

**Background:** Gastric cancer remains a leading global health challenge, despite advances in surgical techniques and perioperative care. Patients with gastric cancer present with a degree of postoperative complications, most notably anastomotic fistulas, which can lead to a high level of morbidity and mortality. Although significant advances have been made in their management by implementing less invasive methods, issues and debate remain regarding their early detection and treatment decisions. The purpose of this study was to emphasize the particularities of the treatment of postoperative fistulas in gastric cancer surgery, focusing on risk factors as well as management strategies. **Methods:** This retrospective study analyzed risk factors, diagnostic methods, and treatment strategies for anastomotic fistulas in 527 patients undergoing curative gastric cancer surgery over the span of five years, highlighting postoperative complication rates, the management of postoperative complications, and the primary risk factors for developing fistulas. **Results:** Conservative treatment combined with minimally invasive interventions achieved a primary success rate of over 65%, with surgical intervention being reserved for severe cases. The primary risk factors identified were an advanced tumor stage, total gastrectomy, type II diabetes mellitus, and a high number of transfusions required, as well as hypoalbuminemia. **Conclusions:** Although further research is required to standardize treatment protocols and reduce the morbidity and mortality associated with postoperative fistulas, understanding the primary elements of its causation can prove helpful in choosing the correct treatment.

## 1. Introduction

Gastric cancer remains one of the most common cancers globally [1], but the mortality rate continues to decline due to advancements in surgical techniques and perioperative management [2]. Currently, radical gastrectomy is still the only potentially curative therapy for resectable gastric cancer. However, surgical treatment includes the standard dissection of the lymph nodes and a variety of reconstructive methods, with the complexity leading to a high risk of postoperative deaths and complications [3]. One of the most dreaded postoperative complications is an anastomotic fistula, which can be life-threatening and is associated with increased treatment costs, prolonged hospitalization, and postoperative mortality [4].

The prevalence and the consequences of the fistula vary depending on the anastomosis site, with incidence rates ranging from 2.7% to 15% [3,4]. Fistulas are the leading cause of mortality after gastrointestinal resections, with mortality rates between 18% and 60% [5].

Identifying risk factors is essential for the prevention and treatment of fistulas. Numerous studies have indicated that tumor location, gender, comorbidities, neoadjuvant therapy, surgical technique, the surgeon’s experience, and multiorgan resection could induce fistula formation. However, it has not yet been established which of the factors are decisive, leading to suboptimal preoperative and postoperative care. Considering the large number of studies about postoperative surgical complications in gastric cancer, the topic remains of great relevance and a source of debate in the literature.

Currently, treatment methods for fistulas are classified into surgical interventions, endoscopic treatment, and conservative strategies. Initially, postoperative fistulas in elective gastric cancer surgery were treated exclusively by surgery. The current trend in managing these fistulas is to combine conservative treatment with minimally invasive approaches, including interventional radiology and/or endoscopy. Conservative treatment consists of fasting, enteral nutrition via a nasojejunal tube and/or parenteral nutrition, fluid–electrolyte balance restoration, and broad-spectrum antibiotics. The standard procedure in specialized centers is the endoscopic placement of a nasojejunal tube under radiologic control.

Upon confirming the presence of an intraperitoneal collection/abscess, the preferred treatment is drainage using a CT or ultrasound-guided pig-tail tube. Combining conservative treatment with nasojejunal feeding tube placement and minimally invasive drainage has shown success rates of up to 81% in specialized studies in the literature [1].

The endoscopic management of anastomotic fistulas through the temporary placement of a self-expandable metallic stent (SEMS) has gained popularity in recent years due to improvements in endoscopic techniques and because it is less invasive than traditional surgery. Studies have reported success rates of 60–90% in closing the fistula after stent placement, with stents typically being maintained for 6–10 weeks to ensure complete closure. These results suggest that six weeks could be enough for the healing of an anastomotic fistula, but in some cases, it is recommended to keep the device for longer to assure the complete closure of the defect. The prolonged in situ placement of a metallic stent can increase the risk of migration, incorporation into the tissue, or aortoesophageal fistulas [6,7]. There is no general consensus on the indications for applying a stent regarding the size of the fistula, the moment of stent application, or the type of fistula.

Authors such as Singh et al. [8] have achieved high healing rates for small postoperative fistulas (under 1 cm) by applying conservative treatment and the image-guided drainage of collection without the need for a stent. In other specialized studies, such as that by Kim et al., a significant healing rate is described for postoperative fistulas under 2 cm through the application of one of the following interventional endoscopic methods: clips, sealing the tract with collagen-based biological products, or self-expandable metal stents [9,10]. The surgical treatment of postoperative fistulas is reserved for patients with severe complications (total anastomotic dehiscence with generalized peritonitis) or cases of conservative treatment failure associated with a minimally invasive approach. Studies in the literature confirm this, with authors like Lang considering that surgical reintervention remains the last resort, given the 64% mortality rate [5]. This underscores the necessity of identifying risk factors, optimal treatment, and the sequencing and feasibility of such treatment in the multimodal management of postoperative fistulas in elective surgery for gastric cancer. Most guidelines are challenged by the same issues, which complicate early diagnosis due to the paucity of symptoms as well as the lack of specificity in current bloodwork [3,4,5].

The aim of this study is to emphasize the particularities of the treatment of postoperative fistulas in gastric cancer surgery, focusing on the risk factors, as well as management strategies.

## 2. Materials and Methods

### 2.1. Patients and Variables Included in the Study

This retrospective study was conducted between January 2016 and December 2020, during which 527 patients were targeted for curative gastric cancer surgery. The hospital’s database was accessed to extract the data of the patients included in the study. The main inclusion criteria for the patients were:

- Curative interventions for gastric cancer;
- Patients over 18 years old;
- Patients who signed informed consent for data processing;
- Elective interventions.

Patients who underwent palliative interventions, had Siewert type II/III adenocarcinoma, refused surgical intervention, had comorbidities contraindicating surgery, did not undergo elective surgery, or did not provide informed consent were excluded from the study. Data were collected retrospectively using a standardized form, which included patient demographic information (sex, age, and environment), diagnostic methods (imaging and endoscopy), medical history (comorbidities such as diabetes, hypertension, history of malignancy, ASA score, and history of neoadjuvant treatment), smoking status, symptoms and their onset, perioperative details (type of intervention, type of resection, type of anastomosis, duration of surgery, blood loss, and multiorgan resections), intraoperative complications, blood transfusion requirements, tumor parameters (histological type and tumor stage), 30-day mortality, and biological parameters (hemoglobin, albumin, and total protein). Data were extracted from the hospital’s database and manually cross-checked with patient charts to complete any missing information. Data curation was performed by the authors listed below.

### 2.2. Types of Surgical Interventions

During the analyzed period, patients included in the study underwent subtotal or total gastrectomies with curative intent, performed by the surgical team at the “Octavian Fodor” Institute of Gastroenterology and Hepatology. These procedures followed the principles of D1+/D2 lymphadenectomy in accordance with established guidelines. The main methods of digestive tract reconstruction included gastro-duodenal anastomosis (Billroth I), gastro-jejunal anastomosis (Billroth II), and gastro-jejunal anastomosis with a Roux-en-Y loop for subtotal gastrectomies, while total gastrectomies involved esophago-jejunal anastomosis with a Roux-en-Y loop. Anastomoses were performed using both manual techniques and circular staplers. When necessary, duodenal stump closure was achieved with a mechanical stapler, with the staple line reinforced by continuous sutures.

### 2.3. Diagnosis of an Anastomotic Fistula

The primary diagnostic methods for anastomotic fistulas include clinical evaluation, biochemical analysis, and conventional imaging techniques such as ultrasound, upper digestive endoscopy, water-soluble contrast radiography, and computed tomography (CT). When an anastomotic fistula was clinically suspected—indicated by symptoms such as abdominal defense response, pathological fluid collection at drainage sites, pronounced febrile syndrome, and inflammatory biochemical markers (leukocytosis, elevated CRP/procalcitonin)—patients were referred to the imaging department for further diagnostic assessment using conventional imaging methods.

### 2.4. Analysis of Patients with Anastomotic Fistulas

Patients were divided into two groups (patients with anastomotic fistulas vs. patients without an anastomotic fistula), and a univariate analysis was performed for all parameters included in the database. Additionally, a separate analysis was conducted for patients with anastomotic fistulas, covering information such as the location of the fistula, the frequency of post-operative fistula occurrence, the initial diagnosis of the fistula, the time interval from diagnosis to fistula closure, the time from fistula diagnosis to discharge, and the closure of the fistula within 30 days post-operation.

### 2.5. Treatment of Anastomotic Fistulas

The majority of patients initially received conservative treatment, which included cessation of oral intake, continuous gastrointestinal decompression via nasogastric tube placement, enteral and parenteral nutritional support, and broad-spectrum antibiotic therapy. In cases of infected collection, intra-abdominal abscess, or hydrothorax, minimally invasive interventions were performed, such as ultrasound- or CT-guided percutaneous drainage with catheter placement. Surgical reintervention was considered for patients experiencing rapid clinical deterioration, including septic syndrome or hemorrhagic shock. When conservative treatment failed or was prolonged without symptom resolution, a multidisciplinary approach was taken to determine interventional treatment. Conservatively treated patients were categorized into two groups—early resolution (Group 1) and late resolution (Group 2)—for univariate and multivariate analysis of risk factors associated with prolonged conservative treatment.

### 2.6. Statistical Analysis

The data were collected using Microsoft Excel 2020, and statistical analysis was performed using IBM SPSS v26.0. The Kolmogorov–Smirnov and Shapiro–Wilk tests were used to assess the normality of the data distribution. For normally distributed quantitative variables, the T-test for independent samples and ANOVA were used to compare means between two or more groups, respectively. For non-normally distributed quantitative variables, the Mann–Whitney U and Kruskal–Wallis tests were used to compare medians between two or more groups. Means were reported with standard deviations, and medians were reported with the interquartile range (IQR). Categorical variables were represented as percentages, and the Chi-Squared and Fisher tests were used for analysis. Statistically significant parameters from the univariate analysis were included in the multivariate analysis using Cox proportional hazards. In multiple comparisons, the statistical significance value was adjusted using Bonferroni correction. The threshold for statistical significance was considered *p* < 0.05.

## 3. Results

### 3.1. General Findings

A total of 527 patients were included in the study. The baseline characteristics of the patients are listed in Table 1. Of these patients, 40 developed postoperative fistulas (anastomotic or duodenal stump fistulas—7.5%). The median age of the patients was 62 years (IQR 54–69), with a BMI of 23.8 (IQR 21.7–25.2). The distribution of patients with comorbidities was relatively uniform (50.3%—254 patients). Approximately 40% of patients (211 patients) were identified as having advanced tumor stage (III). One in four patients received neoadjuvant therapy (136—25.8%). About two-thirds of the patients underwent subtotal gastrectomy (326—61.9%), while the rest were eligible for total gastrectomy. All patients who underwent total gastrectomy received an esophagojejunostomy (201—38.1%). The most commonly used anastomosis in subtotal gastrectomies was gastro-duodenal anastomosis (153—29.1%). Multiorgan resection was performed in 53 cases (10.1%). The 30-day mortality rate was 2.4% (13 cases).

### 3.2. Risk Factors in the Development of Anastomosis Fistula

A univariate analysis of the variables included in the study was performed to highlight the risk factors in anastomotic fistulas. Table 2 includes the analysis of demographic parameters as well as the patients’ comorbidities.

Anastomotic fistulas were identified in older patients (median 63 IQR 56.5–70 vs. median 58 IQR 52.5–66, *p* = 0.001), as well as in patients with comorbidities (10.1% vs. 5.1%, *p* = 0.02), particularly those with diabetes mellitus (10.9% vs. 5.7%, *p* = 0.03). Statistically significant differences were also recorded in patients with a higher ASA score (26.3%, ASA 4, and 13.1%, ASA 3, *p* = 0.002). The distribution of neoadjuvant treatment was heterogeneous, with no statistically significant differences (8.1% vs. 7.4%, *p* = 0.79). Slightly significant differences were observed in female patients (5.1% vs. 9.4%, *p* = 0.06). There were no differences regarding smoking status (7.8% vs. 7.5%, *p* = 0.9) or weight status (median BMI 23.9 IQR 21.4–25.3 vs. 23.6 IQR 22.0–25.1, *p* = 0.34).

The analysis of preoperative characteristics between the two groups, as shown in Table 3, identifies a statistically significant difference in preoperative median hemoglobin levels (9.9 IQR 7.5–11.2 vs. 11.0 IQR 9.6–12.4, *p* = 0.001), albumin levels (3.3 IQR 2.6–3.8 vs. 3.7 IQR 2.9–4.1, *p* = 0.001), and total protein levels (6.1 IQR 5.5–7 vs. 6.9 IQR 6.2–7.4, *p* = 0.001). The occurrence of anastomotic fistulas is statistically significantly associated with the presence of symptoms for more than 6 months (12.5% vs. 3.9%, *p* = 0.001). There are no statistically significant differences in symptom specificity between the two groups (7.4% vs. 7.7%, *p* = 0.87).

Regarding intraoperative characteristics between the two groups, statistically significant differences were observed in the type of total resection, which was associated with a higher risk of anastomotic fistulas compared to subtotal resection (11.4% vs. 5.2%, *p* = 0.008). Moreover, the esojejunal anastomosis was the most frequently involved in fistula formation, being significantly associated compared to gastro-duodenal anastomosis, gastro-jejunal Billroth II, and excluded loop anastomosis (11.4% vs. 5.2% vs. 4.9% vs. 3.3%, *p* = 0.002). Statistically significant differences were also noted in multiorgan resections (15.1% vs. 6.7%, *p* = 0.02), as well as in cases requiring a higher number of transfusions, compared to cases requiring fewer than two transfusions (12.7% vs. 4.3%, *p* = 0.002). There was no statistically significant difference regarding operative time over 180 min (9.5% vs. 5.3%, *p* = 0.06).

No statistically significant differences were found regarding the method of anastomosis (8.2% manual vs. 6.4% mechanical, *p* = 0.45) or the presence of intraoperative incidents (12% vs. 7.4%, *p* = 0.39). The intraoperative characteristics and the differences between the two groups are summarized in Table 4.

In Table 5, the postoperative parameters and the histopathological analysis of the two groups are highlighted. There are statistically significant differences regarding the median duration of hospitalization (21 days IQR 16–27 vs. 8 days IQR 6.5–10.5, *p* = 0.001) and 30-day mortality (12.5% vs. 1.6%, *p* = 0.001). Patients with anastomotic fistulas present a significantly more advanced tumor stage compared to early stages (11.3% vs. 4.5% vs. 6.1%, *p* = 0.02). No statistically significant differences were observed regarding advanced histological grade (10% vs. 5.2%, *p* = 0.11).

Significant parameters identified in the univariate analysis (age over 60, ASA score over 3, presence of comorbidities, presence of diabetes mellitus, multiorgan resection, type of resection, type of anastomosis, advanced tumor stage, hemoglobin below 10.5 g/dL, albumin below 3.5 g/dL, total protein below 6.5 g/dL, late onset of symptoms, hospitalization duration over 12 days, transfusion requirement over 2 units, and diabetes mellitus) were included in the multivariate analysis, with the significance threshold adjusted using Bonferroni correction (significance threshold alpha = 0.05/13 = 0.0038).

The statistically significant factors in the multivariate analysis are highlighted in Table 6. The main significant factors identified are the type of resection (total gastrectomy, HR = 2.93, 95% CI—1.45–7.12, *p* = 0.001), type of anastomosis (esojejunal anastomosis, HR = 2.97, 95% CI—1.97–7.11), albumin below 3.5 g/dL (HR = 3.67, 95% CI—1.97–5.34, *p* = 0.001), transfusion requirement over 2 units (HR = 1.97, 95% CI—1.11–6.27, *p* = 0.003), and diabetes mellitus (HR = 2.44, 95% CI—1.09–4.11, *p* = 0.001).

### 3.3. Diagnosis and Characteristics of Fistulas

The median onset of fistulas is 7 days (IQR 5.5–11) according to the data presented in Table 7. Two-thirds of the diagnosed fistulas occurred more than 7 days after surgery (27 patients, 67.5%), while only two patients developed a fistula within 3 days after surgery (5%).

The main characteristics regarding the diagnostic method for fistulas, as well as the time intervals from diagnosis to closure and from diagnosis to discharge, are highlighted in Table 8. More than half of the patients were diagnosed via computed tomography (21/40, 52.5%), with the smallest proportion diagnosed based on clinical appearance (7/40, 17.5%). The median time from diagnosis to fistula closure was 18 days (IQR 11–24). Closure of the fistula within 30 days after surgery occurred in 32 patients (82.5%).

The main locations of fistulas are represented in Table 9. Following diagnosis through imaging methods, the localization was quantified. The most common site was the esophago-jejunal anastomosis (14 patients, 35%), followed by the duodenal stump fistula (10 patients, 25%) and the gastro-duodenal anastomosis (8 patients, 20%). One case of a fistula at the excluded loop of the Roux Y configuration was reported, along with three cases of complex fistulas, where at least two fistula sites were identified (7.5%).

### 3.4. Multimodal Treatment of Postoperative Fistulas

Seven patients underwent surgical intervention as a first-line treatment due to early fistula onset or rapid clinical–biological deterioration.

In 33 patients (82.5%), a combined conservative and minimally invasive interventional approach was attempted. Among these 33 patients, 11 subsequently required surgical intervention. The success rate for the interventional approach combined with conservative treatment was 66.7% (22 patients). A mortality rate of 12.5% (5 patients) was recorded.

In the interventional approach, 12 patients were drained via ultrasound guidance (52.2%), while 11 cases were treated endoscopically (47.8%), including 5 patients who were stented, 5 patients with pigtail drainage, and 1 patient with an endo-sponge.

Of the 11 patients who underwent surgery, 6 received suture reinforcement of the anastomosis or duodenal stump along with improved drainage. Four patients underwent reconstruction of the anastomosis, while one patient had closure of the esophageal and jejunal stumps with a jejunostomy for feeding and drainage.

In order to identify risk factors that may lead to the prolongation of non-surgical treatment in anastomotic fistulas, a univariate analysis was performed. Patients treated non-surgically were divided into two groups (under 18 days vs. over 18 days) as shown in Table 10.

Statistically significant differences between the two groups were observed in the median albumin values (3.4 g/dL IQR 3.1–3.7 vs. 2.9 g/dL IQR 2.4–3.5, *p* = 0.001). Additionally, significantly lower differences were recorded in the median total protein values (6.0 g/dL IQR 5.7–6.5 vs. 5.5 g/dL IQR 5.1–5.9, *p* = 0.001). From the perspective of fistula location, statistically significant differences were noted in esophagojejunal location (87.5% vs. 12.5%, *p* = 0.001). Patients with delayed healing had a significantly older median age (62.5 years IQR 54–59 vs. 67 years IQR 61–72, *p* = 0.03). Furthermore, a significant association between delayed healing and the presence of diabetes mellitus was highlighted (80% vs. 20%, *p* = 0.02). No statistically significant differences were observed in other variables.

Statistically significant parameters from the univariate analysis were included in the multivariate analysis, with adjustment of the statistical significance threshold, as shown in Table 11.

The main statistically significant factors were albumin levels below 3 g/dL (HR = 3.11, 95% CI—1.74–9.12, *p* = 0.01) and the presence of diabetes mellitus (HR = 1.98, 95% CI—1.05–5.22, *p* = 0.01).

## 4. Discussion

The variability of postoperative complications in gastric cancer is influenced by the complexity of surgical intervention and strict adherence to gastrointestinal anastomosis techniques. According to the literature, gastrointestinal fistulas—particularly esophagojejunal—significantly increase mortality rates [11,12]. In this study, the incidence of postoperative fistulas was 7.5%, aligning with reported rates in the specialized literature. Mortality among patients with postoperative fistulas was 12%, whereas recent studies suggest lower rates, ranging from 2% to 8% [13,14,15]. This discrepancy may be attributed to late presentation, with symptoms appearing more than six months after surgery. Additionally, over 40% of patients who developed fistulas in our study had advanced disease (Stage III). Patients in Stage IV were excluded, which may contrast with studies reporting lower mortality rates, where most patients present at earlier stages [12,14,15]. The prevalence of comorbidities in our study was higher than in other studies, with diabetes mellitus being the most common. Consequently, diabetes was analyzed separately. This finding aligns with the existing literature, where Chan et al. suggest that type II diabetes lasting over 10 years is a risk factor for anastomotic fistula development [14]. In this study, diabetes mellitus was identified as a risk factor both for fistula formation and for delayed non-surgical resolution.

Advanced age is also considered a risk factor for anastomotic fistulas. Although recent studies emphasize a biological frailty index where age is less influential [16], surgical eligibility is primarily determined by preoperative parameters independent of age [17]. Our findings suggest that age over 60 is a significant risk factor for anastomotic fistula development. This is further supported by the statistical significance of an ASA score above 3, with nearly 40% of patients with fistulas falling into this category.

Patients included in the study had a body mass index (BMI) at the lower limit of the overweight range. Although no significant differences were observed between the two groups, the combination of BMI and marked hypoproteinemia may suggest an increased incidence of sarcopenia with developed visceral fat. This has been identified as a risk factor for postoperative complications in gastrointestinal surgery [14,18]. Sex variability may also play a role, as men tend to accumulate more visceral fat. While visceral fat can be correlated to some extent with BMI—a parameter analyzed in this study—no association was found between sex and fistula development, nor was visceral fat specifically assessed. Further studies are needed to explore this relationship. Hemoglobin variation is extensively studied in this context and is considered one of the most significant risk factors for anastomotic fistula development in digestive tract surgery [19,20,21]. It often results from occult blood loss, either preoperatively or intraoperatively due to recorded blood loss. Our study reinforces this, particularly in the multivariate analysis, where patients who received more than two intraoperative blood transfusions had a significantly higher risk of developing fistulas.

A possible explanation for the lack of statistical significance in hemoglobin values may be the chronic nature of preoperative anemia in some patients. Those experiencing significant intraoperative blood loss may have a higher risk of fistula development compared to patients with pre-existing anemia but controlled blood loss. Further studies are needed to explore this association in greater detail.

Protein status is a crucial factor in the development of fistulas in digestive surgery, as inadequate nutritional resources can impair postoperative healing, as suggested by current studies [15,20]. This paper examines hypoproteinemia from two perspectives. First, it is considered both an independent and cumulative risk factor for anastomotic fistula development. Second, hypoproteinemia—particularly hypoalbuminemia—is identified as an independent and cumulative risk factor for prolonged non-surgical fistula treatment. These findings highlight the potential need for more aggressive enteral and parenteral nutritional support during the perioperative and postoperative periods. From an intraoperative perspective, factors such as total gastrectomy, the need for more than two transfusions, esophagojejunal anastomosis, and multiorgan resections are considered independent risk factors for anastomotic fistula development. The complexity of total gastrectomy with esophagojejunal anastomosis is widely documented in the literature, with particular emphasis on the surgeon’s experience and the quality of the tertiary center. However, as this is a retrospective analysis, assessing these factors is challenging and introduces a potential data collection bias.

Additionally, our study may present a causality bias between the type of intervention and the nature of the anastomosis, as esophagojejunal anastomosis was the only method used in total gastrectomy procedures. Nonetheless, total gastrectomy was associated with a nearly threefold higher risk of fistula development compared to subtotal gastrectomy (HR = 2.93, 95% CI: 1.45–7.12), a finding supported by the current literature. Mbulaiteye et al. also reported significant differences in fistula rates between total and proximal gastrectomy (2.3–4.6%) [13].

An important consideration in multiorgan resections is the additional physiological stress, which is often linked to hypoproteinemia. Further studies are needed to explore this association.

Early fistula formation (within 72 h) is considered a technical defect, with current studies recommending surgical intervention in such cases [22]. In this study, two early fistulas were identified and successfully treated with surgical reintervention. The majority of fistulas (approximately 70%) were late-onset, likely influenced by the previously discussed risk factors. The median time to fistula formation was seven days after surgery, consistent with findings in the specialized literature [13,14,15,20].

Although more than 50% of the fistulas in this study were diagnosed via computed tomography, imaging indications were based on thorough clinical evaluation. In three patients who underwent primary surgical intervention, clinical presentation alone was sufficient to confirm the presence of a fistula, eliminating the need for additional imaging. Fistula closure within 30 days after surgery was achieved in over 80% of cases, aligning with the reported literature [17,19].

Esophago-jejunal and duodenal stump fistulas remain a diagnostic and treatment challenge, being associated with a significantly higher mortality rate compared to other fistula locations. Due to the small sample size and the limited number of deaths attributed to fistulas, a statistical analysis could not be carried out in this regard. However, our study aligns with the literature, highlighting the high morbidity and mortality in these two locations [13,15].

There is no established consensus on the optimal method or timing for transitioning from conservative to interventional or surgical treatment in anastomotic fistulas. Management primarily focuses on achieving spontaneous healing of the anastomotic defect through conservative, interventional, or surgical techniques while preventing intraperitoneal contamination and minimizing intra-abdominal fluid collection.

Conservative treatment is particularly important in cases of non-infected collection, reducing additional physiological stress. In our study, the success rate of conservative treatment was 63% (10/16), consistent with the current literature [14]. Although the endo-sponge is considered the gold standard in fistula management [15,22], its use in our study was limited due to high costs at the time. Despite the staged nature of fistula treatment, surgery remains a last-resort option, as it is associated with significantly higher mortality rates compared to other treatment approaches [18,23].

The median time from fistula diagnosis to closure was 18 days (IQR 11–24), while the median time from diagnosis to discharge was 22 days (IQR 15–29), results that are comparable to or slightly better than those reported in the literature [24].

In cases of prolonged conservative treatment, several independent risk factors were identified, including median age (62.5 years vs. 67 years), albumin and total protein levels, and the presence of diabetes mellitus. Additionally, delayed resolution was more common in esophago-jejunal anastomosis fistulas, likely due to the poor vascularization and friability of the esophageal anastomotic partner, which impairs healing. In contrast, gastro-jejunal anastomosis fistulas demonstrated earlier resolution, benefiting from a better vascularized and more resilient anastomotic partner, with greater resistance to ischemia and easier technical mobilization, thereby reducing tension at the anastomotic site. One of the most important aspects remains the early detection of the fistula. Having multiple minimally invasive methods as well as conservative treatment options can improve the outcome and increase the survival in fistula treatment. Recent studies have shown that textural analysis as well as radiomics can detect intraperitoneal infected fluid collection at a significantly higher rate, which can improve early detection of postoperative fistulas [25,26].

This study has several limitations, primarily due to its retrospective design. Data collection bias is a potential concern, as reliance on database records may lead to transcription inconsistencies. Additionally, the sample size is relatively small compared to other studies in the specialized literature. The heterogeneity between the two groups (fistula vs. non-fistula) may also be influenced by the use of data from a single tertiary center (Regional Institute of Gastroenterology and Hepatology “O. Fodor” Cluj-Napoca).

Moreover, there is no standardized consensus in the literature regarding the definition, diagnosis, and treatment algorithm for postoperative fistulas in gastric surgery. Patient follow-up is further complicated by non-compliance, making long-term outcome assessment challenging. Despite these limitations, our findings align with the existing literature regarding key risk factors, particularly poor preoperative nutrition and diabetes mellitus. While other studies have identified respiratory issues, obesity, and chronic renal failure as risk factors [27], these were not significant in our study, likely due to the specific characteristics of our patient cohort.

Furthermore, although we identified primary risk factors for prolonged anastomotic leakage, research on optimal treatment strategies remains limited. Some studies have proposed various surgical techniques to minimize anastomotic leakage risk, but their efficacy has yet to be fully evaluated [26,27].

## 5. Conclusions

Despite the multimodal approach to managing esophago-jejunal fistulas, these conditions remain associated with high morbidity. Several risk factors contribute to the prolonged course of conservative treatment. Surgical intervention is typically reserved for early fistulas and cases that are refractory to non-surgical treatments, particularly when there is significant clinical and biological deterioration. The implications of this study suggest that optimizing perioperative management could improve patient outcomes, emphasizing the need for careful monitoring and tailored interventions. Furthermore, the impact of surgical center quality and surgeon experience plays a crucial role in the management of fistulas and should be considered when developing treatment strategies.

## Figures and Tables

**Table 1:** Basic parameters of the study.

Parameters		Values
Age (years)		62 (54–69)
BMI (kg/m^2^)		23.8 (21.7–25.2)
Gender	Male	295 (55.9%)
	Female	232 (44.1%)
ASA Score	1	219 (41.5%)
	2	228 (43.2%)
	3	61 (11.5%)
	4	19 (3.6%)
Comorbidities	Absent	262 (49.7%)
	Present	254 (50.3%)
Neoadjuvant treatment	Without	391 (74.2%)
	With	136 (25.8%)
Multiorgan resection	Without	474 (89.9%)
	With	53 (10.1%)

**Table 2:** Analysis of demographic factors and personal medical history of the sample according to the two groups.

Parameter		Fistula (n = 40)	Without Fistula (n = 487)	*p* Value
Age (years)		63 (56.5–70)	58 (52.5–66)	0.001
BMI (kg/m^2^)		23.9 (21.4–25.3)	23.6 (22.0–25.1)	0.34
Gender	Male	28 (70%)	267 (54.8%)	0.06
	Female	12 (30%)	220 (45.2%)	
ASA Score	1	11 (27.5%)	208 (42.7%)	0.002
	2	16 (40%)	212 (43.5%)	
	3	8 (20%)	53 (10.9%)	
	4	5 (12.5%)	14 (2.9%)	
Comorbidities		26 (65%)	229 (47.1%)	0.02
Smoker status		14 (35%)	166 (34.1%)	0.9
Diabetes mellitus		20 (50%)	164 (33.6%)	0.33
Neoadjuvant treatment		11 (8.1%)	125 (91.9%)	0.79

**Table 3:** Analysis of preoperative characteristics of patients based on the two groups.

Parameter		Fistula (n = 40)	Without Fistula (n = 487)	*p* Value
Median hemoglobin (g/dL)		9.9 (7.5–11.2)	11.0 (9.6–12.4)	0.001
Median albumin (g/dL)		3.3 (2.6–3.8)	3.7 (2.9–4.1)	0.001
Median total protein (g/dL)		6.1 (5.5–7)	6.9 (6.2–7.4)	0.001
Symptom onset	Under 6 months	12 (30%)	291 (59.7%)	0.001
	Over 6 months	28 (70%)	196 (40.3%)	
Symptoms	Nonspecific	17 (42.5%)	201 (41.2%)	0.87
	Specific	23 (57.5%)	286 (58.8%)	

**Table 4:** Analysis of intraoperative characteristics of patients based on the two groups.

Parameter		Fistula (n = 40)	Without Fistula (n = 487)	*p* Value
Duration of the intervention	Under 180 min	13 (32.5%)	231 (47.4%)	0.06
	Over 180 min	27 (67.5%)	256 (52.6%)	
Median Intraoperative blood loss (mL)		185 (100–225)	148 (130–250)	0.14
Transfusion requirement	<2 units	13 (32.5%)	301 (61.8%)	**0.002**
	2 or more	27 (67.5%)	186 (38.2%)	
Type of resection	Total	23 (57.5%)	178 (36.5%)	**0.008**
	Subtotal	17 (42.5%)	309 (63.4%)	
Type of anastomosis	Esophago-jejunal	23 (57.5%)	178 (36.5%)	**0.002**
	Gastro-duodenal	8 (20%)	145 (29.7%)	
	Gastro-jejunal (Billroth II)	5 (12.5%)	78 (16.0%)	
	Gastro-jejunal (Roux-en-Y)	4 (10%)	86 (17.6%)	
Performing the anastomosis	Mechanically	12 (30%)	175 (35.9%)	0.45
	Manually	28 (70%)	312 (64.1%)	
Multiorgan resection	Without	32 (80%)	442 (90.7%)	**0.02**
	With	8 (20%)	45 (9.3%)	
Intraoperative incidents	Without	37 (92.5%)	465 (95.5%)	0.39
	With	3 (7.5%)	22 (4.5%)	

Bolded values are statistically significant ones (*p* < 0.05).

**Table 5:** Analysis of postoperative characteristics of patients based on the two groups.

Parameter		Fistula (n = 40)	Without Fistula (n = 487)	*p* Value
Histological type	G1–G2	14 (5.2%)	253 (94.8%)	0.11
	G3–G4	26 (10%)	234 (90%)	
Tumor stage	I	6 (6.1%)	92 (93.9%)	**0.02**
	II	10 (4.5%)	208 (95.5%)	
	III	24 (11.3%)	187 (88.7%)	
30-day mortality		5 (12.5%)	8 (1.6%)	**0.001**
Median duration of hospitalization		21 (16–27)	8 (6.5–10.5)	**0.001**

Bolded values are statistically significant ones (*p* < 0.05).

**Table 6:** Multivariate analysis for identifying clinicopathological factors associated with anastomotic fistulas.

Parameter		Hazard Ratio (95% CI)	*p* Value
Age over 60		1.41 (1.12–8.34)	0.18
ASA score above 3		1.68 (1.09–12.14)	0.11
Comorbidities		1.36 (1.01–6.87)	0.23
Multiorgan resection		1.55 (1.31–5.27)	0.07
Total gastrectomy (type of resection)		2.93 (1.45–7.12)	**0.001**
Type of anastomosis	Eso-jejunal	2.97 (1.97–7.11)	**0.001**
	Gastro-duodenal	2.3 (1.14–5.72)	
	Gastro-jejunal (Billroth II)	1.12 (0.98–2.41)	
	Gastro-jejunal (Roux-en-Y)	1	
Stage III tumor		1.83 (1.26–5.17)	0.04
Hemoglobin below 10.5 g/dL		2.01 (1.45–6.18)	0.01
Albumin below 3.5 g/dL		3.67 (1.97–5.34)	**0.001**
Total protein below 6.5 g/dL		3.01 (1.44–7.11)	0.004
Late onset of symptoms		1.77 (1.01–8.72)	0.11
Hospitalization longer than 12 days		2.88 (1.01–11.45)	0.24
Transfusion requirement above 2 units		1.97 (1.11–6.27)	**0.003**
Diabetes mellitus		2.44 (1.09–4.11)	**0.001**

Bolded values are statistically significant ones (*p* < 0.05).

**Table 7:** Interval of occurrence and median onset duration of fistulas.

Interval Between Intervention and Fistula		Frequency
Frequency of fistula occurrence	Under 3 days	2 (5%)
	3–7 days	11 (27.5%)
	Over 7 days	27 (67.5%)
Median onset of fistula		7 (5.5–11)

**Table 8:** Diagnostic methods and time intervals (diagnosis to closure, diagnosis to discharge, closure within 30 days) in postoperative fistulas.

Parameter		Frequency
Initial diagnosis of a fistula	Clinical–biological	7/40 (17.5%)
	CT	21/40 (52.5%)
	Radiology + water-soluble contrast	12/40 (20%)
Time from diagnosis to fistula closure		18 (11–24)
Time from diagnosis to patient’s discharge		22 (15–29)
Closure of the fistula within 30 days after surgery		33/40 (82.5%)

**Table 9:** Main fistula sites.

Fistula Site	Frequency
Gastro-duodenal anastomosis	8 (20%)
Esophago-jejunal anastomosis	14 (35%)
Gastro-jejunal anastomosis	4 (10%)
Duodenal stump	10 (25%)
Limb of the jejunal loop	1 (2.5%)
Complex fistulas	3 (7.5%)

**Table 10:** Univariate analysis of risk factors for prolonged non-surgical treatment duration.

Parameter		Under 18 Days (n = 8)	Over 18 Days (n = 14)	*p* Value
Median age		62.5 (54–59)	67 (61–72)	**0.03**
Sex	M	5 (62.5%)	8 (57.1%)	0.8
	F	3 (37.5%)	6 (42.9%)	
BMI		23.4 (21.6–25.4)	23.6 (22.1–25.4)	0.91
ASA Score	Under 3	6 (75%)	4 (28.5%)	0.35
	Over 3	2 (25%)	10 (71.5%)	
Hemoglobin		9.6 (8.9–10.3)	9.2 (8.6–9.8)	0.06
Albumin		3.4 (3.1–3.7)	2.9 (2.4–3.5)	**0.001**
Total protein		6.0 (5.7–6.5)	5.5 (5.1–5.9)	**0.001**
Diabetes mellitus		3 (37.5%)	12 (85.7%)	**0.02**
Smoker status	Smokers	3 (30%)	7 (70%)	0.57
Neoadjuvant treatment		2 (25%)	6 (75%)	0.4
Type of resection	Total	5 (62.5%)	9 (64.3%)	0.93
	Subtotal	3 (37.5%)	5 (35.7%)	
Duration of the intervention	Under 180 min	2 (25%)	6 (42.8%)	0.4
	Over 180 min	6 (42.8%)	8 (57.2%)	
Transfusion requirement	Under 2 units	3 (37.5%)	3 (21.4%)	0.12
	Over 2 units	5 (62.5%)	11 (78.6%)	
Fistula site	Gastro-duodenal	2 (25%)	2 (14.2%)	**0.001**
	Esophago-jejunal	1 (12.5%)	7 (50%)	
	Gastro-jejunal	4 (50%)	0 (0%)	
	Duodenal stump	1 (12.5%)	3 (21.4%)	
	Complex fistulas	0 (0%)	2 (14.2%)	

Bolded values are statistically significant ones (*p* < 0.05).

**Table 11:** Multivariate analysis of risk factors for prolonged non-surgical treatment duration (Bonferroni correction, alpha = 0.05/4 = 0.0125).

Parameter	Hazard Ratio (95% CI)	*p* Value
Age over 65	1.67 (1.11–8.14)	0.04
Albumin under 3	3.11 (1.74–9.12)	**0.01**
Total protein under 5.7	2.77 (1.63–11.09)	0.06
Fistula site (esophago-jejunal)	2.19 (1.14–10.87)	0.1
Diabetes mellitus	1.98 (1.05–5.22)	**0.01**

Bolded values are statistically significant ones (*p* < 0.05).

## Data Availability

Data are available upon request.
